# Iron therapy effect on chronic heart failure and iron-deficiency anemia: a systematic review and meta-analysis

**DOI:** 10.1186/s43044-024-00545-8

**Published:** 2024-08-30

**Authors:** Yusra Pintaningrum, Ni Putu Yunandari

**Affiliations:** 1 Cardiology and Vascular Department, Faculty of Medicine and Health Science, Mataram University, Mataram, Indonesia; 2 Faculty of Medicine and Health Science, Mataram University, Mataram, Indonesia

**Keywords:** Oral iron therapy, Intravenous iron therapy, Functional capacity, Hospitalization risk, Mortality risk, Chronic heart failure, Iron-deficiency anemia

## Abstract

**Background:**

Heart failure complicated with iron deficiency is associated with impaired functional capacity, poor quality of life, increased hospitalization, and mortality. This systematic review and meta-analysis were conducted to assess the effect of oral and intravenous iron therapy on functional capacity, hospitalization risk, and mortality risk in patients with chronic heart failure and iron-deficiency anemia.

**Methods:**

Search for published scientific articles using the PRISMA (Preferred Reporting, Items for Systematic Reviews and Meta-Analysis) method conducted on Cochrane Library, PubMed Central, and Medline databases published in the last 20 years. Further systematic review and meta-analysis using RevMan version 5.4 were performed based on the included published scientific articles.

**Results:**

Based on the meta-analysis of included studies, the analytical results of intravenous iron therapy in patient with chronic heart failure and iron-deficiency anemia showed there is 30.82 (MD = 30.82: 95% CI 18.23–43.40) meter change in patient 6MWT, there is likelihood of 0.55 times (55%) (RR = 0.45: 95% CI 0.30–0.68) lower risk of hospitalization and lower risk of mortality (RR = 0.18: 95% CI 0.04–0.78), because heart failure worsening both with statistically significant overall effect compared with placebo.

**Conclusions:**

There is statistically significant effect of intravenous iron therapy to improve patient functional capacity and reduce likelihood of hospitalization risk of 0.55 times (55%) in patient with chronic heart failure and iron-deficiency anemia.

## Background

Anemia defined as hemoglobin count <12 g/dl in women and <13 g/dl in men, iron deficiency is one of the most common nutritional deficiencies that can trigger anemia, affecting one-third of world general population and also can be found as comorbidity in several chronic disorders [1]. Iron deficiency is increasingly recognized as an important comorbidity in heart failure patient and can be found up to 35–50% of patients with heart failure [1–3]. Mechanisms underlying the development of iron deficiency in heart failure still not fully investigated yet, but iron deficiency may be a consequence of impaired iron absorption, augmented gastrointestinal loss, and reduced availability of utilizable iron from the reticuloendothelial system [2,4,5].

It is also possible that the presence of some comorbidity such as anemia could be an important contributing factor in the poor outcome of heart failure patient [6] Prevalence of iron deficiency in heart failure is related to the severity of the disease and is a strong and independent predictor of patients outcome [2] Iron plays an important role in oxygen transport, not only through hematopoiesis but also in the metabolism of cardiac and skeletal muscles. All these factors play a role in the reduced exercise capacity in heart failure [2] Patients with iron-deficiency anemia and chronic heart failure are characterized by high mortality and increased rates of urgent hospitalizations due to heart failure worsened progression [4]

Heart failure complicated with iron deficiency is associated with impaired functional capacity, poor quality of life, and increased mortality [1,4]. Recently, several studies have investigated the effects of intravenous iron therapy in iron-deficient patients with heart failure, including the FAIR-HF [7], CONFIRM-HF [3], EFFECT-HF [2], also there are IRONOUT-HF [8] that try to confirmed oral iron therapy as inexpensive and more accessible to some patient. The gathered experimental and clinical data provided evidence to consider iron deficiency as a potential therapeutic target in patients with heart failure [1,4]. This study aim to summarize and analyze the available clinical data in the form of meta-analysis to investigate the effect of intravenous or oral iron therapy to functional capacity, hospitalization risk, and mortality risk of patient with chronic heart failure and iron-deficiency anemia.

## Methods

**Fig. 1:**
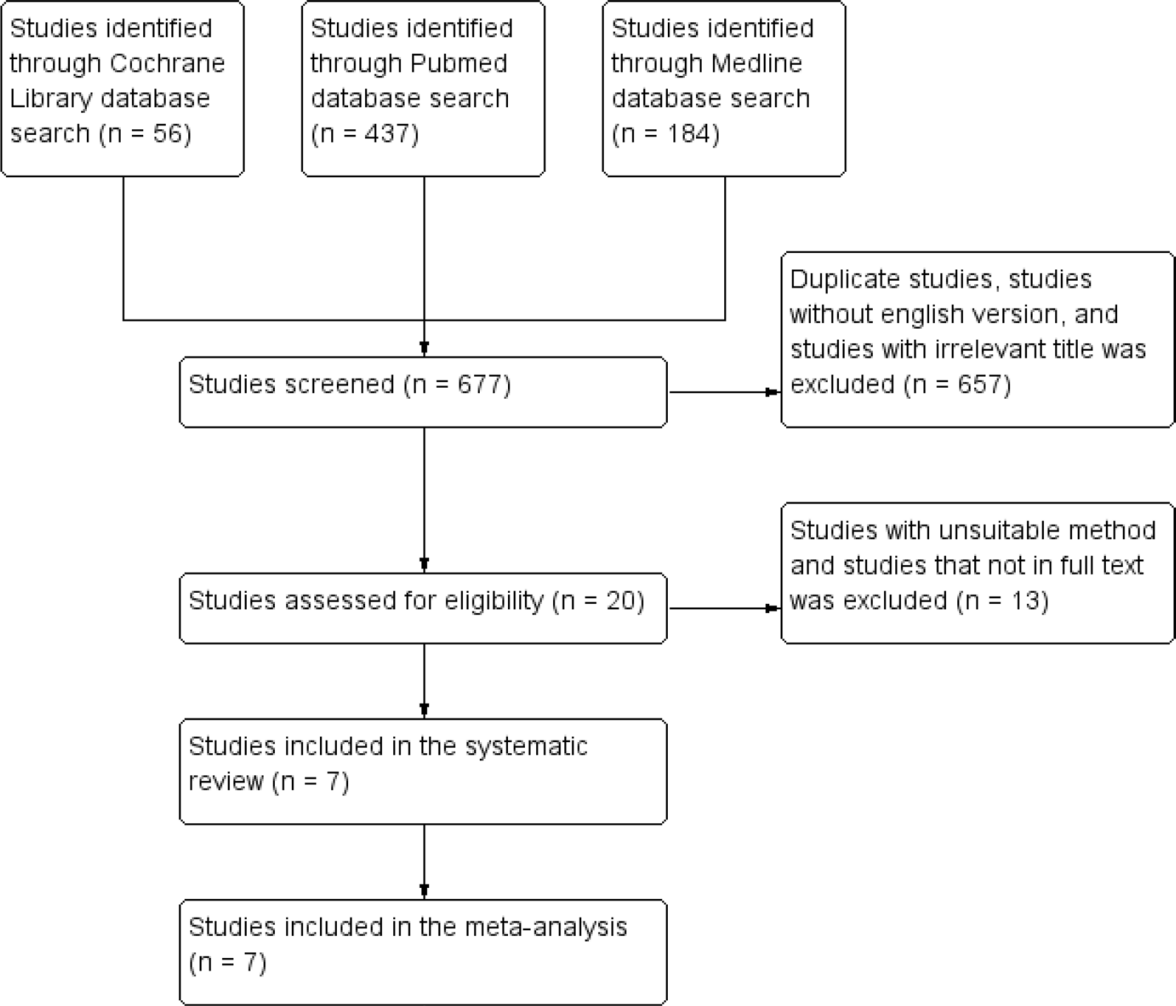
PRISMA method diagram in choosing relevant studies sources

The current review and analysis refer to statements in the instruction of the Preferred Reporting Items for Systematic Review and Meta-Analysis (PRISMA) (Fig. 1). A detailed protocol has been registered earlier in PROSPERO.

### Data sources and search strategy

The method used to construct systematic review and meta-analysis is PRISMA (Preferred Reporting, Items for Systematic Reviews, and Meta-Analysis). To obtain studies for this review, we searched PubMed Central, PubMed NCBI, and Cochrane Library using the following keywords: “iron therapy effect” AND “functional capacity” AND “hospitalization” AND “mortality” AND “chronic heart failure” AND “iron-deficiency anemia.”

### Study selection and quality assessment

We select the latest 20-year quantitative study that compared oral and intravenous iron therapy with placebo for at least 12 weeks on chronic heart failure (NYHA Class II–III or LV ejection fraction < 50%) and iron-deficiency anemia (Hb < 12,5 g/dL for men and Hb < 11,5 g/dL for woman and serum ferritin < 100 ug/L) patient. Iron therapy included in this study are IV iron sucrose or IV ferric carboxymaltose and oral iron polysaccharide or oral ferrous sulfate compared with placebo to assessed functional capacity (6MWT), hospitalization rate, or mortality of the patient. The research samples in the included research studies were patients with heart failure regardless of the cause, whether valvular heart disease, ischemic cardiomyopathy, or dilated cardiomyopathy.

This initial search yielded 677 hits, of which 657 were studies with irrelevant titles, duplicate studies, studies without English versions, and studies with unavailable full text. The remaining 20 studies were further assessed if they met specific inclusion criteria for the purpose of this analytical review, which 12 studies were not met the inclusion criteria in this study. The final sample included consisted of 7 relevant studies that have a representative purpose with this analytical review. For quality assessment of the study, we manually assess (1) risk of bias, (2) blinding in the studies, (3) loss of data, (4) selective report of the study result, (5) arrangement construction, (6) study personnel, (7) result analysis method, and (8) alternative source of bias in the study.

### Data synthesis and statistical analysis

Data from the studies included in this review are combined to estimate mean difference of oral iron therapy and IV iron therapy functional capacity, and further estimate hospitalization rate and mortality risk ratio of iron therapy compared with placebo for at least 12 weeks on chronic heart failure and iron-deficiency anemia patient.

Based on the I^2^ of each outcome, a summary effect measure was conducted. Statistical analysis was conducted using Mantel and Haenszel’s method [9]. The patients were regarded as a single unit of analysis to investigate functional capacity mean difference of Oral and IV Iron Therapy effect against placebo, further followed by risk ratio analysis of hospitalization rate and mortality of the patient. The mean difference (MR) and risk ratios (RRs) with a 95% confidence interval (CI) [9]. The heterogenic nature of the trials was evaluated using the Q and I^2^ analysis methods. The Q analysis show heterogeneity if *P*-value <0.05 is defined statistically significant and the I^2^ values >70%. *I*^2^ value of 25%, 50%, and 75% were defined as low, moderate, and high levels of heterogeneity, respectively [10]. If the *I*^2^ value being <70% and *P*-value >0.05 is defined as statistically insignificant heterogeneity, the outcome analyzed with fixed effect model, but if *I*^2^ value being >70% or *P*-value >0.05 is defined as statistically significant heterogeneity, the outcome analyzed with random-effect model and progressed to subgroup analysis [10] Sensitivity analysis performed by excluding each study if *I*^2^ >75% or *P* > 0.1. Funnel plot symmetry was assessed visually and any other bias of the study sources assessed manually. The data were processed utilizing Review Manager software (Review Manager (RevMan) [Computer program]. Version 5.4. The Cochrane Collaboration, 2020.).

## Results

### Demographic characteristics of the included studies

We identified 677 studies from the keywords, and after screening and eligibility assessment of those studies, we found 7 potentially relevant studies with the purpose of this analytical review. All of the potentially relevant studies were Randomized Controlled Trial, at least 12 weeks studies, placebo controlled, consist of 5 double blinded studies and 1 single blinded studies. Total participant of these studies are 1009 participants from 21 countries all across the world include Argentina, United Kingdom, Poland, Germany, Italy, Spain, Greek, Sweden, Norway, Switzerland, Russia, Poland, Ukraine, Austria, Portugal, Australia, Belgium, France, Netherlands, Indonesia, and USA. The demographic characteristic and functional capacity (6MWT) outcome are summarized in Table 1, hospitalization and mortality outcome of the included studies are summarized in Table 2.

**Table 1:** Demographic characteristic and functional capacity (6MWT) outcome of the included studies

Studies	Study location	Study method	Study duration (weeks)	Blinding	Placebo controlled	Participants (n)	Outcome		
							Placebo 6MWT change	6MWT change	
								IV iron therapy	Oral iron therapy
Anker et al., 2009^7^ (FAIR-HF)	Germany, Italy, Spain, Greek, Sweden, Norway, Switzerland, Russia, Poland, England, Ukraine	RCT	24	Double Blind	Yes	402	10.2 ± 66.67 (n = 134)	38.6 ± 75.38 (n = 268)	–
Ponikowski et al., 2015^3^ (CONFIRM-HF)	Austria, Italy, Poland, Portugal, Russia, Spain, Sweden, United Kingdom, Ukraine	RCT	52	Double Blind	Yes	246	-21.8 ± 106.5 (n = 121)	16.6 ± 98.04 (n = 125)	–
Lewis et al., 2017^8^ (IRONOUT-HF)	United States	RCT	16	Double Blind	Yes	225	32.0 ± 34 (n = 114)	–	19.0 ± 32 (n = 111)
Suryani et al., 2017^11^	Indonesia	RCT	12	Double Blind	Yes	41	− 16.84 ± 40.05 (n = 19)	–	63.07 ± 11.87 (n = 22)
Total						914			

Abbreviation: RCT, Randomized controlled trial; 6MWT, 6-min walk test; IV, Intravenous

**Table 2:** Demographic characteristic, hospitalization, and mortality outcome of the included studies

Studies	Study location	Study method	Study duration (weeks)	Blinding	Placebo controlled	Participants (n)	Outcome			
							Hospitalization		Mortality	
							Placebo	IV iron therapy	Placebo	IV iron therapy
Tobli et al., 2007^6^	Argentina	RCT	24	Double blind	Yes	40	5/20	0/20	-	-
Okonko et al., 2008^1^ (FERRIC-HF)	United Kingdom, Poland	RCT	16	Single Blind	No	35	1/11	1/24	0/11	1/24
Anker et al., 2009^7^ (FAIR-HF)	Germany, Italy, Spain, Greek, Sweden, Norway, Switzerland, Russia, Poland, United Kingdom, Ukraine	RCT	24	Double Blind	Yes	459	9/154	7/305	3/154	0/305
Ponikowski et al., 2015^3^ (CONFIRM-HF)	Austria, Italy, Poland, Portugal, Russia, Spain, Sweden, United Kingdom, Ukraine	RCT	52	Double Blind	Yes	301	32/151	10/150	3/151	4/150
Van Veldhusen et al., 2017^2^ (EFFECT-HF)	Australia, Belgium, France, Germany, Italy, Netherlands, Poland, Russia, Spain	RCT	24	Single Blind	No	174	13/86	13/88	4/86	0/88
Total						1009	65/422	35/587	13/402	5/567

Abbreviation: RCT, Randomized controlled trial; 6MWT, 6-min walk test; IV, Intravenous

### Outcome characteristics of the included studies

Total participant in 7 studies included are 1009 participants. Among these 1009 participants, there are 914 participants complete functional capacity assessment with 6MWT (6-minute walk test), 388 (42.5%) participants taken placebo, 393 (43.0%) participants taken IV iron therapy, and 133 (14.5%) participants taken oral iron therapy. Outcome characteristics of functional capacity included in this study are summarized in Table 1.

All of the participants complete hospitalization assessment and 969 participants complete mortality assessment. In the placebo group, there are 65 (15.40%) participants hospitalized due to heart failure out of 422 participants, and there are 13 (3.23%) death participants due to heart failure worsening out of 402 participants that taken placebo. In the IV iron therapy group, there are 35 (5.96%) participants hospitalized due to heart failure out of 587 participants, and there are 5 (0.88%) death participants due to heart failure worsening out of 567 participants that taken IV iron therapy. Outcome characteristics of hospitalization and mortality in this study are summarized in Table 2.

### Oral iron therapy compared against placebo for functional capacity (6MWT) change on chronic heart failure and iron-deficiency anemia patient

**Fig. 2:**
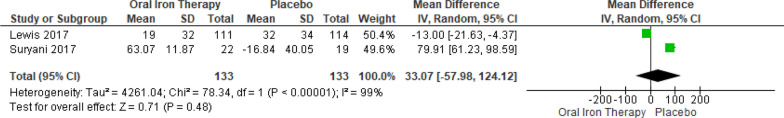
The forest plot of oral iron therapy compared against placebo for functional capacity (6MWT) change on chronic heart failure and iron-deficiency anemia patient

In the meta-analysis forest plot presented in Fig. 2 showed heterogeneity model analysis (*I*^2^= 99%; *X*^2^ = 4261.04; *P* = <0.00001). Based on the heterogeneity analytical result, data compiled from two studies included were statistically heterogeneous, on random-effect analysis, we conducted a summary effect measure to evaluate the mean difference of functional capacity change outcome from oral iron therapy compared against placebo. The analytical results showed there is 33.07 (MD = 33.07: 95% CI − 57.98 to 124.12) change in mean difference with statistically insignificant overall effect (*P* = 0.48) of oral iron therapy compared with placebo. This result showed there are no significant effect of iron therapy usage compared with placebo to improve functional capacity of chronic heart failure and iron-deficiency anemia patient.

### Intravenous iron therapy compared against placebo for functional capacity (6MWT) change on chronic heart failure and iron-deficiency anemia patient

**Fig. 3:**
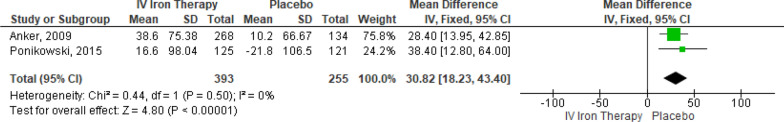
The forest plot of intravenous iron therapy compared against placebo for functional capacity (6MWT) change on chronic heart failure and iron-deficiency anemia patient

In the meta-analysis, forest plot presented in Figure 3 showed heterogeneity model analysis (*I*^2^= 0%; *X*^2^ = 0.44; *P* = 0.50). Based on the heterogeneity analytical result, data compiled from two studies included were statistically homogenous, so we conducted a summary effect measure to evaluate the mean difference of functional capacity (6MWT) change outcome from intravenous iron therapy compared against placebo. The analytical results showed there is 30.82 (MD = 30.82: 95% CI 18.23–43.40) change in mean difference with statistically significant overall effect (*P* < 0.00001) of intravenous iron therapy compared with placebo to change functional capacity of the patient. This result showed there is a significant higher likelihood on functional capacity improvement on patient upon usage of intravenous iron therapy compared against placebo.

### Intravenous iron therapy compared against placebo for hospitalization risk on chronic heart failure and iron-deficiency anemia patient

**Fig. 4:**
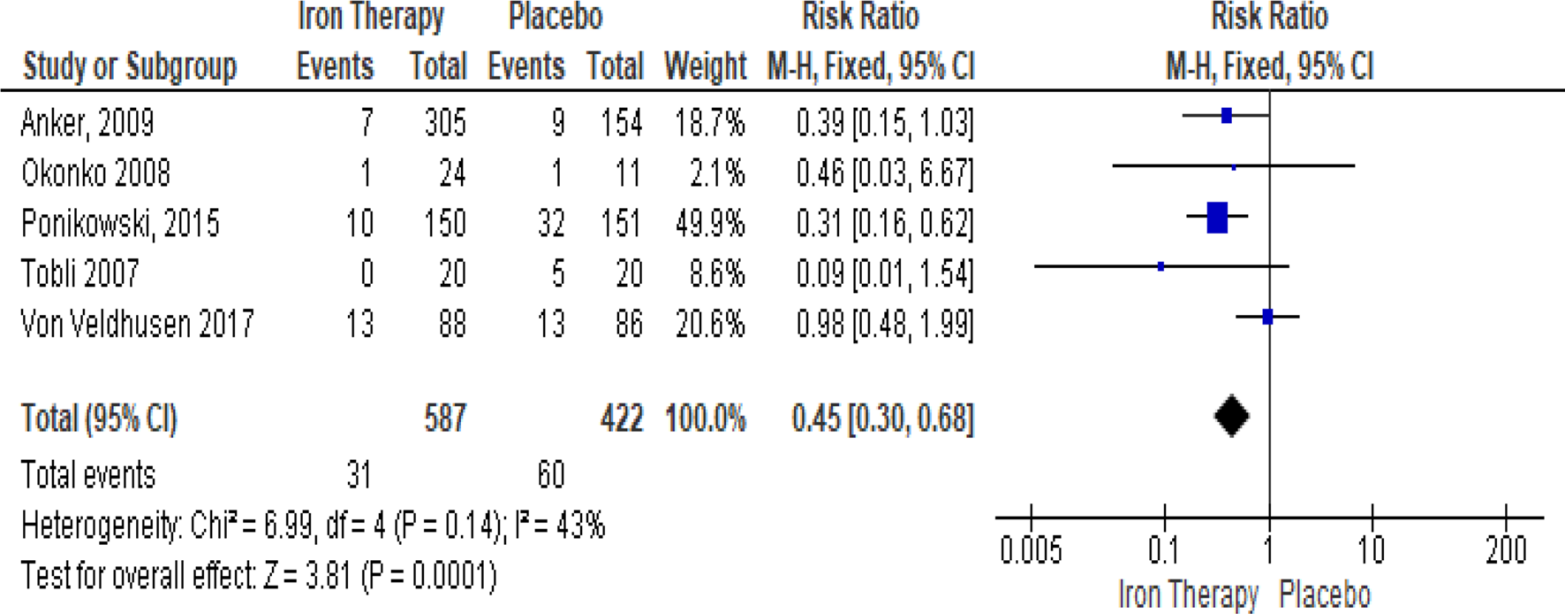
The forest plot of intravenous iron therapy compared against placebo for hospitalization risk on chronic heart failure and iron-deficiency anemia patient

In the meta-analysis, forest plot presented in Fig. 4 showed heterogeneity model analysis (*I*^2^= 43%; *X*^2^ = 6.99; *P* = 0.14). Based on the heterogeneity analytical result, data compiled from five studies included were statistically homogenous, so we conducted a summary effect measure to evaluate the hospitalization risk outcome from intravenous iron therapy compared against placebo. The analytical results showed there is 0.45 (RR = 0.45: 95% CI 0.30–0.68) times risk ratio with statistically significant overall effect (*P* = 0.00001) of intravenous iron therapy compared with placebo. This result showed there is a significant lesser likelihood of hospitalization risk on patient upon usage of intravenous iron therapy compared against placebo.

### Intravenous iron therapy compared against placebo for mortality risk on chronic heart failure and iron-deficiency anemia patient

**Fig. 5:**
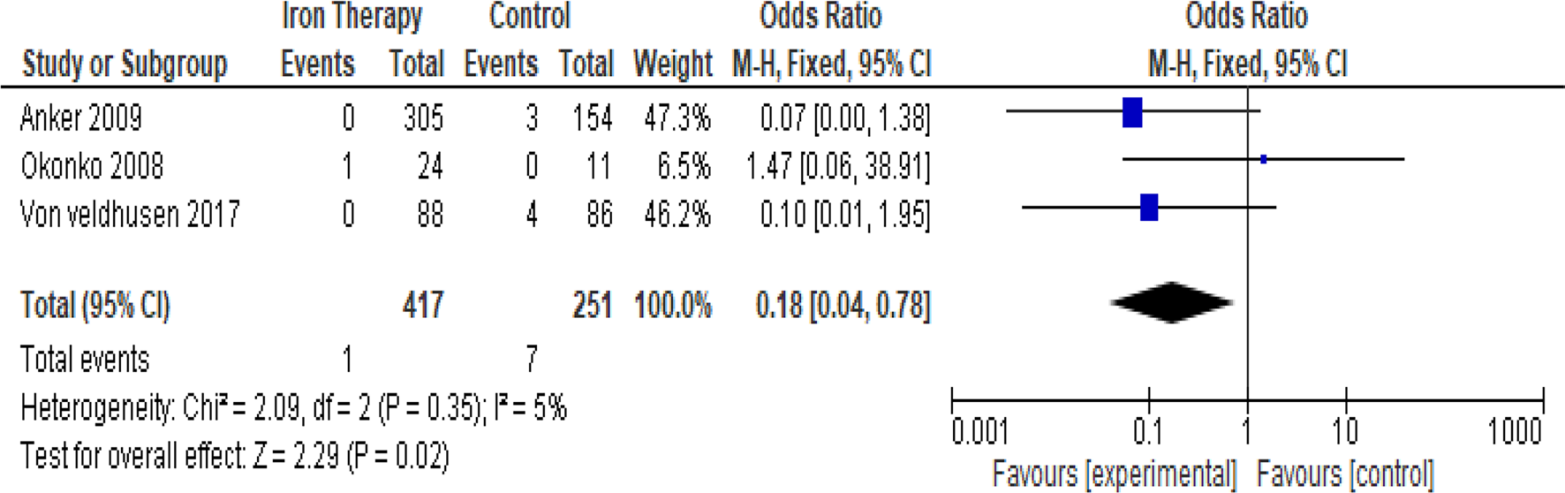
The forest plot of intravenous iron therapy compared against placebo for mortality risk on chronic heart failure and iron-deficiency anemia patient

In the meta-analysis, forest plot presented in Fig. 5 showed heterogeneity model analysis (*I*^2^= 5%; *X*^2^ = 5.00; *P* = 0.35). Based on the heterogeneity analytical result, data compiled from four studies included were statistically homogenous, so we conducted a summary effect measure to evaluate the mortality risk outcome from intravenous iron therapy compared against placebo. The analytical results showed there is 0.18 (RR = 0.18: 95% CI 0.04–0.78) times risk ratio with statistically insignificant overall effect (*P* = 0.02) of intravenous iron therapy compared with placebo. This result showed there is significant effect of intravenous iron therapy usage compared with placebo to reduce mortality risk of chronic heart failure and iron-deficiency anemia patient.

## Discussion

Heart failure complicated with iron deficiency is associated with impaired functional capacity, poor quality of life, and increased mortality [1,4] We investigate clinical data from seven included studies to summarize and analyze oral and intravenous iron therapy effect on functional capacity, hospitalization risk, and mortality risk of patient with chronic heart failure and iron-deficiency anemia.

In this meta-analysis, we analyzed two studies about oral iron therapy and two studies about intravenous iron therapy compared with placebo effect on 6MWT (6-minute walking test) change as patient functional capacity assessment. Our meta-analysis showed there is statistically insignificant overall effect (*P* = 0.48) in oral iron therapy compared with placebo to improve patient functional capacity, but intravenous iron therapy showed statistically significant overall effect (*P* < 0.00001) compared with placebo to improve patient functional capacity. Intravenous iron therapy showed 6MWT change mean difference around 30.82 m compared with patient that received placebo. Our findings further strengthened recommendation for screening and intravenous iron treatment for patient with iron-deficiency anemia and chronic heart failure to improve patient quality of life that also stated in another studies with same findings like FAIR-HF [7], CONFIRM-HF [3], and EFFECT-HF [2] studies.

In this meta-analysis, we also further investigate intravenous iron therapy effect in hospitalization risk and mortality risk of chronic heart failure and iron-deficiency anemia patient. To achieve our goal, we include five studies for hospitalization risk assessment and four studies for mortality risk assessment. Our meta-analysis of intravenous iron therapy showed statistically significant overall effect (*P* < 0.00001) compared with placebo to reduce the likelihood of patient hospitalization risk because of worsening heart failure by 0.55 times (55%). Our meta-analysis also analyzed intravenous iron therapy effect in mortality risk.

Intravenous iron therapy showed there is statistically significant overall effect (*P* = 0.02) in intravenous iron therapy compared with placebo to reduce the likelihood of mortality risk in chronic heart failure and iron-deficiency anemia. Our study also strengthened the recommendation to give intravenous iron therapy for patient with iron-deficiency anemia and chronic heart failure to improve functional capacity and reduced likelihood of hospitalization risk because of worsening heart failure as stated in other studies include FAIR-HF [7], CONFIRM-HF [3], and EFFECT-HF [2].

In this study construction, we also find there is still so few study about oral iron therapy effect to patient functional capacity. We also found out there is still no study about oral iron therapy effect to hospitalization risk and mortality risk of chronic heart failure and iron-deficiency anemia. Because of the expensiveness and low availability of intravenous iron therapy in low-middle income countries, further investigation of oral iron therapy still needed at the moment.

## Conclusions

Findings in this study showed there is statistically significant effect of intravenous iron therapy to improve patient functional capacity and reduce likelihood of hospitalization risk of 0.55 times (55%) in patient with chronic heart failure and iron-deficiency anemia. These findings strengthened the benefit of iron deficiency screening and intravenous therapy recommendation on chronic heart failure and iron-deficiency anemia patient. We also found there is still lack of clinical data about oral iron therapy effect in patient with chronic heart failure and iron-deficiency anemia, so further investigation still needed in this topic at the moment.

## Data Availability

Not applicable.
